# Physician characteristics associated with proper assessment of overstated conclusions in research abstracts: A secondary analysis of a randomized controlled trial

**DOI:** 10.1371/journal.pone.0211206

**Published:** 2019-01-25

**Authors:** Yasushi Tsujimoto, Takuya Aoki, Kiyomi Shinohara, Ryuhei So, Aya M. Suganuma, Miho Kimachi, Yosuke Yamamoto, Toshi A. Furukawa

**Affiliations:** 1 Department of Healthcare Epidemiology, School of Public Health in the Graduate School of Medicine, Kyoto University, Kyoto, Japan; 2 Department of Nephrology and Dialysis, Kyoritsu Hospital, Kawanishi, Hyogo, Japan; 3 Department of Health Promotion and Human Behavior Kyoto University Graduate School of Medicine / School of Public Health, Kyoto, Japan; University of California San Diego, UNITED STATES

## Abstract

**Objectives:**

Little is known about the physician characteristics associated with appraisal skills of research evidence, especially the assessment of the validity of study methodology. This study aims to explore physician characteristics associated with proper assessment of overstated conclusions in research abstracts.

**Design:**

A secondary analysis of a randomized controlled trial.

**Setting and participants:**

We recruited 567 volunteers from the Japan Primary Care Association.

**Methods:**

Participants were randomly assigned to read the abstract of a research paper, with or without an overstatement, and to rate its validity. Our primary outcome was proper assessment of the validity of its conclusions. We investigated the association of physician characteristics and proper assessment using logistic regression models and evaluated the interaction between the associated characteristics and overstatement.

**Results:**

We found significant associations between proper assessment and post-graduate year (odds ratio [OR] = 0.67, 95% confidence interval [CI] 0.49 to 0.91, for every 10-year increase) and research experience as a primary investigator (PI; OR = 2.97, 95% CI 1.65 to 5.34). Post-graduate year and PI had significant interaction with overstatement (*P* = 0.015 and < 0.001, respectively). Among participants who read abstracts without an overstatement, post-graduate year was not associated with proper assessment (OR = 1.04, 95% CI 0.82 to 1.33), and PI experience was associated with lower scores of the validity (OR = 0.58, 95% CI 0.35 to 0.96).

**Conclusion:**

Physicians who have been in practice longer should be trained in distinguishing overstatements in abstract conclusions. Physicians with research experience might be informed that they tend to rate the validity of research lower regardless of the presence or absence of overstatements.

**Trial registration:**

UMIN000026269.

## Introduction

Evidence-based medicine (EBM), or evidence-based practice (EBP), has been recognized as a key skill for healthcare workers [1, 2]. There are five steps to practicing EBM: 1) formulation of clinical uncertainty into an answerable question; 2) systematic retrieval of best evidence available; 3) critical appraisal of evidence for validity, clinical relevance, and applicability; 4) application of results in practice; and 5) evaluation of performance [1, 3]. However, critical appraisal skills are limited among physicians [4]. A previous systematic review reported there are various barriers to primary care physicians’ use of EBM [5]. For example, they lack time to find the evidence or access to journals, or lack the knowledge to appraise it. Therefore, EBM is not well established among primary care physicians.

Moreover, a considerable number of reports of randomized controlled trials (RCTs) draw conclusions in their abstracts that are inconsistent with their study findings [6]. Boutron et al. suggested that more than half of the abstracts or main texts of RCT reports with no significant results for any primary outcomes showed inconsistency between the results and the conclusions in their abstracts, and called such inconsistency a “spin” [6]. Our previous study also showed that approximately one third of psychiatry studies claiming effectiveness in their abstract conclusions were exaggerated in comparison with the full text results [7]. Such inappropriate reporting in the abstract, that may be called spin or overstatement, can influence physicians’ interpretation if their critical appraisal skills are limited [6, 7]. Boutron et al conducted an RCT and found that the researchers who read an abstract with a spin were more likely to think that the intervention was beneficial for the patients than those who read an abstract without a spin [8]. Meanwhile, in our previous study, we assessed the influence of overstatements in abstract conclusions in a web-based RCT and showed that primary care physicians make a sound judgment on the validity of abstracts regardless of the presence/absence of overstatements in abstract conclusions when the primary outcomes are appropriately reported in the methods and results sections. A further question is, what are the physician characteristics associated with proper or improper assessment when physicians read abstracts with overstatement. This study therefore aimed i) to explore physician characteristics that were associated with proper assessment of the validity of overstated abstract conclusions, and ii) to investigate whether or not the influence of those characteristics differed depending on the presence or absence of overstatement.

## Materials and methods

The Do Overstated Conclusions Trick Our Readers? (DOCTOR) study is a double-blind RCT that was conducted between January and February 2017. The methods of this study and its primary results have been published elsewhere [9] and are only briefly described here.

### RCT procedure

We recruited volunteers from members of the Japan Primary Care Association (JPCA) by e-mailing invitations. The JPCA was founded in 2010 to promote primary care specialty in Japan, and now boasts of over 10,000 physician members [10, 11]. A total of 567 primary physicians who met the following eligibility criteria were enrolled in this RCT: a member of the JPCA, medical doctor currently in clinical practice, clinical practice experience of more than two years and access to up-to-date clinical research knowledge (we asked how they learn about the recent clinical trials; individuals who did not respond with any information source to this question were excluded). We excluded those who work at research laboratories or educational institutions.

All of the participants provided written informed consent, and the protocol was approved by the Ethical Committee of the Kyoto University Graduate School of Medicine and was prospectively registered with the University Hospital Medical Information Network-Clinical Trial Registry (UMIN000025317).

After registering baseline characteristics, participants were randomly allocated to a research abstract with or without overstatements. An overstatement was defined as ‘inconsistency between the results of primary outcomes in full-text and those deduced from the abstract conclusion’ [7]. We selected four abstracts from PubMed as prototypical examples of overstatement. When the abstract failed to report the study findings appropriately, we supplemented information about their primary outcome(s) to the methods and results section of the research abstract, so that participants who had critical appraisal skills and who were allocated to overstated abstracts could directly check the inconsistency between the results and conclusions in the abstract. S1 Text shows the text of the abstracts used in the DOCTOR study. All participants were asked to score the validity of the conclusion in the given abstract: ‘How valid is this conclusion, in your opinion, on a scale of 0 to 10, with 0 being not at all and 10 being very likely’.

### Primary outcome for the present study

Our primary outcome was proper assessment of the validity of the abstract conclusion. We defined proper assessment as scoring less than five for abstracts with an overstated conclusion, and scoring five or more for abstracts without an overstated conclusion. This cut-off was based on the mean score of all the participants’ ratings in the original RCT.

### Physician characteristics

We assessed the following physician characteristics: sex, post-graduate year, any board certification, doctorate degree, work place (hospital or clinic), clinical research experience as a principal investigator (PI), the number of abstracts read in the last month (0–4 or 5 ≥), previous attendance at an EBM workshop, and access to research information (only from a pharmaceutical company or not).

### Statistical analysis

#### We conducted two statistical analyses

Analysis 1: Characteristics of primary care physicians associated with proper assessment when they read overstated abstract conclusions.

Participants who were allocated to abstracts with overstated conclusions were analysed. We first summarised physician characteristics. Then, we explored characteristics associated with proper assessment using Fisher’s exact test and a multivariate logistic regression model for univariable and multivariable analysis, respectively.

Analysis 2: Examining whether or not the influence of the identified characteristics was dependent on the presence or absence of overstatement.

Participants in both groups (with and without overstatement) in the RCT were assessed. The characteristics identified by statistical Analysis 1 were analysed using multivariable logistic regression model with the addition of the interaction with overstatement. We performed a subgroup analysis according to overstatement when the interaction was statistically significant.

Continuous variables were expressed as means (standardized deviation) and categorical variables as numbers (%). A two-sided *P*-value smaller than 0.05 was considered statistically significant. Stata/SE, version 14.0 (StataCorp, College Station, TX, USA) was used for all analyses.

## Results

**Fig 1:**
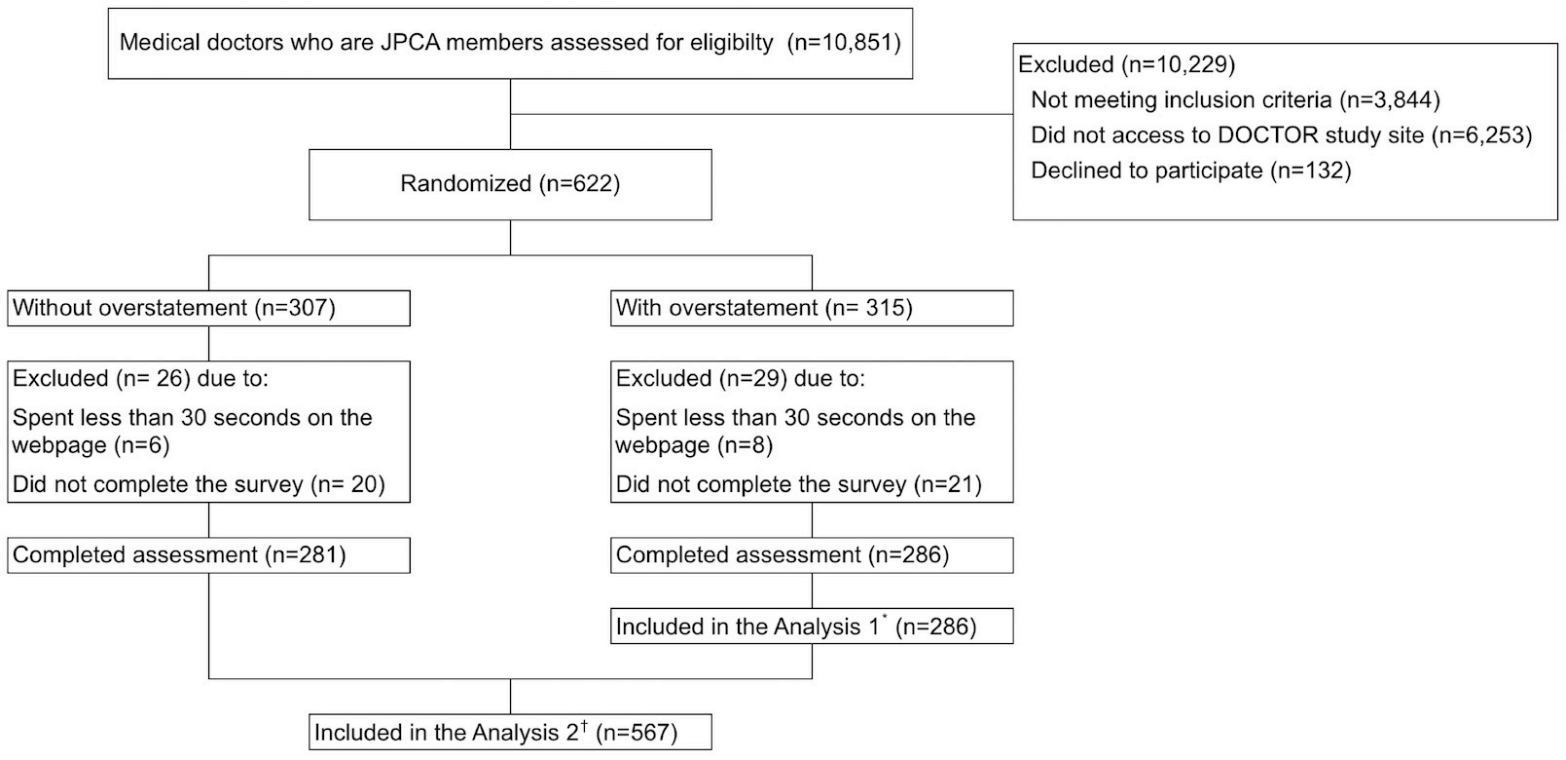
Flow diagram of participants. *Analysis 1: Analysis for Characteristics of primary care physicians associated with proper assessment of overstated abstract conclusions ^†^Analysis 2: Analysis examining whether or not the associated characteristics identified by Analysis 1 were dependent on the influence of overstatement.

Fig 1 shows the flow diagram of the present study.

We sent e-mail invites to 7040 JPCA members, of whom 622 were eligible and subsequently randomly allocated to ‘without overstatement’ (n = 307) and ‘with overstatement’ (n = 315) groups. Fifty-five individuals were excluded because they either spent less than 30 seconds on the webpage (n = 14) or did not complete the survey (n = 41). Most participants read and rated the abstract within four minutes (median time: 162 seconds, 25 seconds per trial; interquartile range [IQR] = 114–236 seconds).

A total of 286 primary physicians in the ‘with overstatement’ group were included in Analysis 1 that examined physicians’ characteristics associated with proper assessment of overstatement. A total of 567 individuals in both groups were included in Analysis 2 that examined whether or not the associated characteristics identified by Analysis 1 were dependent on overstatement.

### Analysis 1: Characteristics of primary care physicians associated with proper assessment for abstracts with overstated conclusion

Table 1 shows the physician characteristics by their assessment results. Post-graduate year, board certification, and PI experience were associated with proper assessment in univariable analyses. Table 2 shows the multivariable analysis for the characteristics associated with proper assessment. Physicians who have been in practice longer gave proper assessment significantly less frequently for abstracts with an overstated conclusion (odds ratio [OR] = 0.67, 95% confidence interval [CI] 0.49 to 0.91 for each 10-year increase). On the other hand, physicians who have experienced clinical research as a PI reported proper assessment significantly more frequently than those who do not have experience as a PI (OR = 2.97, 95% CI 1.65 to 5.34).

**Table 1:** Physician characteristics classified by proper assessment of overstated conclusion.

Category	Characteristics	Proper^†^ (n = 159)		Not proper^‡^ (n = 127)		*p-*value*
Sex, n (%)	Male	133	(83.6)	110	(86.6)	0.51
Postgraduate year, mean (SD)		15.8	(9.5)	19.4	(8.6)	0.001
Workplace, n (%)	Clinic	42	(26.4)	38	(29.9)	0.6
Board certification^§^, n (%)		138	(86.8)	121	(95.3)	0.015
Doctorate grade, n (%)		48	(30.2)	45	(35.4)	0.38
PI^||^, n (%)		71	(44.7)	35	(27.6)	0.003
Information resource, n (%)	Pharmacological company^§^	10	(6.3)	14	(11.0)	0.2
EBM workshop**, n (%)		107	(67.3)	79	(62.2)	0.38
Abstract ≥5^††^, n (%)		92	(57.9)	82	(64.6)	0.27

*p-value for Fisher’s Exact test

^†^Rating less than 5 for the validity of overstated abstract conclusion on a scale of 0 to 10, with 0 being not at all and 10 being very likely

^‡^Rating 5 or more for the validity of overstated abstract conclusion on a scale of 0 to 10, with 0 being not at all and 10 being very likely

^§^Any board certification

^||^Clinical research experience as a principal investigator

^¶^Access to research information (only from pharmacological company)

**Ever attended an evidence based medicine workshop

^††^Reading 5 or more abstracts in the last month

SD = Standardized deviation, PI = Principal investigator, EBM = Evidence based medicine

**Table 2:** Association between physician characteristics and proper assessment of overstated conclusion (n = 286).

Category	Characteristics	OR	95% CI	*p-*value*
Sex	Male	0.74	0.36–1.53	0.42
Postgraduate year	every 10-year increase	0.67	0.49–0.91	0.01
Workplace	Clinic	0.94	0.54–1.66	0.84
Board certification^†^		0.39	0.14–1.04	0.06
Doctorate grade		0.8	0.44–1.45	0.46
PI^‡^		2.97	1.65–5.34	<0.001
Information resource	Pharmacological company^§^	0.67	0.27–1.69	0.40
EBM workshop^||^		0.93	0.54–1.60	0.78
Abstract ≥5^¶^		0.89	0.52–1.54	0.67

*p-value for multivariable logistic regression model

^†^Any board certification

^‡^Clinical research experience as a principal investigator

^§^Access to research information (only from pharmacological company)

^||^Ever attended an evidence based medicine workshop

^¶^Reading 5 or more abstracts in the last month

OR = Odds ratio, CI = Confidence interval PI = Principal investigator, EBM = Evidence based medicine

### Analysis 2: Examining whether or not the influence of the identified characteristics was dependent on the presence or absence of overstatement

In response to the results of Analysis 1, the interactions between overstatement and post-graduate year and PI experience were examined using multivariable logistic regression model. We found a statistically significant interaction between overstatement and both post-graduate year (a 10-year increase; OR = 0.64, 95% CI 0.45 to 0.92, *P* = 0.015) and PI experience (OR = 3.73, 95% CI 1.63 to 3.87, *P* < 0.001).

S1 Table shows the characteristics classified by their assessment among participants who were allocated to abstracts without overstatements. Table 3 shows the subgroup analysis according to the presence or absence of overstatement. The association between post-graduate year and proper assessment was not statistically significant when participants read the abstracts without overstatement (a 10-year increase; OR = 1.04, 95% CI 0.82 to 1.33). The association between PI experience and proper assessment showed opposite directions in the presence or absence of overstatement. Among ninety-four participants with PI experience, forty-seven (50%) provided proper assessment; of one hundred and eighty-seven participants without PI experience, one hundred and twelve (63%) provided proper assessment. PI experience was associated with less proper assessment when they read abstracts without an overstated conclusion (OR = 0.58, 95% CI 0.35 to 0.96).

**Table 3:** Physician characteristics associated with proper assessment, classified by the presence or absence of overstatement (n = 567).

Subgroups		OR	95% CI	*p-*value*
Abstracts without overstatement (n = 281)				
PI^†^		0.58	0.35–0.96	0.034
Postgraduate year	every 10-year increase	1.04	0.82–1.33	0.73
Abstracts with overstatement (n = 286)				
PI^I^		2.72	1.59–4.64	<0.001
Postgraduate year	a 10-year increase	0.58	0.44–0.76	<0.001

*p-value for logistic regression model

^†^Clinical research experience as a principal investigator

OR = Odds ratio, CI = Confidence interval PI = Principal investigator

## Discussion

### Summary

We found statistically significant associations of post-graduate year and PI with proper assessment when the participants read abstracts with overstated conclusions. The effect of both post-graduate year and PI, and proper assessment was different if an overstatement was present or not. Physicians who have been in practice for a long time assessed the validity of the abstracts more properly: they rated the abstracts’ validity lower only when they noticed an overstatement. Meanwhile, the association between PI and proper assessment when the participants read abstracts without overstated conclusions was in the opposite direction: compared with physicians without PI experience, physicians with PI experience were less likely to assess the validity properly; that is, they rated these abstracts’ validity low, even when the abstracts did not contain overstatements.

### Strengths and limitations of the present study

To our knowledge, this is the first study to explore the characteristics of primary care physicians who had good critical appraisal skills for overstated abstracts. We targeted physicians who can access and use up-to-date evidence. The results suggest that overstatement may mislead physicians for whom more time has elapsed since their graduation. In Japan, the Model Core Curriculum, which aimed to standardize education in medical schools, was proposed in 2001 [12]. The EBM concept was introduced across the country and became compulsory for medical students [13]. Our results may reflect this transition in that younger physicians have better critical appraisal skills than older physicians in the country.

Another important finding is that physicians with experience in clinical research may tend to rate the validity of an abstract low even without the presence of overstatements, and are also less likely to be affected by overstatement. A qualitative study found that those with research experience are less likely to find original work confusing and would feel more confident that they can assess research evidence [14]. The confidence in critical appraisal might affect these physicians in a negative way. They might be overly strict to rate any abstract as ‘valid.’ There may be a need for education for researchers with a focus on appraising evidence more fairly.

Our study has several limitations to be considered. First, the participants may not be representative of the JPCA members. The response rate of 11.1% (787/7040) limits the generalizability of our findings. Those who responded to our invitation were potentially avid readers of research evidence, which is the reason why they participated in this study, and therefore they have better critical appraisal skills than other JPCA members. Secondly, we only assessed physicians’ skills to assess the validity of abstracts. This is one aspect of critical appraisal skill. However, physicians rarely read full texts thoroughly [15]. Clinicians often read only the abstract of a journal article because of lack of time and the difficulties of accessing the full article [5, 8]. Further research measuring other aspects of critical appraisal skill may confirm our findings. Lastly, as this study was exploratory in nature, future studies are needed to confirm our results.

### Comparison with existing literature

Many studies have assessed the relationship between clinical experience and quality of health care or mortality [16–18]. They reported that physicians who have been in practice longer might be at risk for lower quality of care; patients treated by older physicians have higher mortality; older physicians have decreased clinical knowledge, low adherence to standards of appropriate treatment and worse performance on process measures of quality with respect to diagnosis, screening and preventive care. Our finding that older physicians have lower critical appraisal skills was consistent with these studies. Decreased critical appraisal skill may be a pathway between clinical experience and worse healthcare outcomes.

A recent study found that compared with family medicine residents who lack research experience, those with research experience mark higher scores in the Fresno test, which is a reliable measure of EBP and critical appraisal of the validity of medical literature [19, 20]. Similarly, our results showed that physicians with PI experience were likely to provide proper assessment when they encounter overstatements, compared with those without PI experience. However, when they encountered appropriately written abstracts, they tended to assess them over-critically. It may be helpful for continued medical education in critical appraisal skills to take such a tendency into account and emphasize that one needs not be too critical in order to assess properly the soundness of evidence.

### Implications for research and practice

Based on our findings, clinicians whose graduation year is old may be a priority population to be alerted about overstatement. This may help prevent worse patient outcomes by older physicians. Previous systematic reviews suggest some effect of teaching EBM to gain critical appraisal skills, however, these studies mainly targeted medical students or residents [21]. Future research should consider the effect of continuing medical education among physicians who have been in practice for a long time. Obviously, researchers should not overstate their findings in the abstract conclusion for the sake of their own research integrity lest they mislead older physicians. Checking the overstatements through the editorial and peer-review process could play an important roll to prevent the production of overstatements.

### Conclusion

Physicians with greater post-graduation years were more likely to be unduly influenced by overstated abstract conclusion. Physicians with clinical research experience as a PI were less likely to be affected by overstatement but this may have been due to the fact that they underrated the validity of study findings regardless of the presence or absence of an overstatement.

### Ethical consideration

The original RCT was approved by the Ethics Committee of Kyoto University Graduate School of Medicine and was conducted in accordance with the Declaration of Helsinki including secondary use of the data. We obtained an online consent of participation from each participant. The protocol of the present study was registered in the University hospital Medical Information Network (UMIN) Clinical Trials Registry (registration number: UMIN000026269).

## Supporting information

S1 Text Full text of five abstracts used in the original RCT. (PDF)

S1 Table Physician characteristics classified by proper assessment of conclusion without overstatement. (PDF)
