# Supplementary material for: Physician characteristics associated with proper assessment of overstated conclusions in research abstracts: A secondary analysis of a randomized controlled trial
Source: PLoS One. 2019 Jan 25;14(1):e0211206. doi: 10.1371/journal.pone.0211206 (PMC6347200; doi:10.1371/journal.pone.0211206)
Supplement: S1 Text — (PDF) [file pone.0211206.s001.pdf]

S1 text: Full text of five abstracts used in the original RCT

We modified abstracts of the following articles.

1. Sternfeld B, Guthrie KA, Ensrud KE, et al. Efficacy of exercise for menopausal symptoms: a randomized controlled trial. Menopause 2014;21(4):330-8.
2. Samus QM, Johnston D, Black BS, et al. A multidimensional home-based care coordination intervention for elders with memory disorders: the maximizing independence at home (MIND) pilot randomized trial. Am J Geriatr Psychiatry 2014;22(4):398-414.
3. Freund-Levi Y, Jedenius E, Tysen-Bäckström AC, Lärksäter M, Wahlund LO, Eriksdotter M. Galantamine versus risperidone treatment of neuropsychiatric symptoms in patients with probable dementia: an open randomized trial. The American journal of geriatric psychiatry 2014;22(4):341-8.
4. Oosterbaan DB, Verbraak MJ, Terluin B, et al. Collaborative stepped care v. care as usual for common mental disorders: 8-month, cluster randomised controlled trial. Br J Psychiatry 2013;203(2):132-9.
5. Lam RW, Parikh SV, Ramasubbu R, et al. Effects of combined pharmacotherapy and psychotherapy for improving work functioning in major depressive disorder. The British Journal of Psychiatry 2013;203(5):358-65.

We added the shaded part to the original abstract.

134

TITLE: Intervention A for menopausal symptoms: a randomized controlled trial

OBJECTIVE: This study aims to determine the efficacy of intervention A for alleviating vasomotor and other menopausal symptoms.

METHODS: Late perimenopausal and postmenopausal sedentary women with frequent vasomotor symptoms (VMS) such as hot flash, sweating, and poor circulation participated in a randomized controlled trial conducted in three sites: 106 women randomized to exercise and 142 women randomized to usual activity. VMS frequency and bother were recorded on daily diaries at baseline and on weeks 6 and 12. Intent-to-treat analyses compared between-group differences in changes in VMS frequency and bother, sleep symptoms (Insomnia Severity Index and Pittsburgh Sleep Quality Index), and mood (Patient Health Questionnaire-8 and Generalized Anxiety Disorder-7 questionnaire). Primary outcomes were VMS frequency and bother mean frequency or bother of VMS at 6 and 12 weeks.

RESULTS: At the end of week 12, changes in VMS frequency in intervention A group (mean change, -2.4 VMS/d; 95% CI, -3.0 to -1.7) and VMS bother (mean change on a four-point scale, -0.5; 95% CI, -0.6 to -0.4) were not significantly different from those in control B group (-2.6 VMS/d; 95% CI, -3.2 to -2.0; P = 0.43; -0.5 points; 95% CI, -0.6 to -0.4; P = 0.75). The exercise group reported greater improvement in insomnia symptoms (P = 0.03), subjective sleep quality (P = 0.01), and depressive symptoms (P = 0.04), but differences were small and not statistically significant when P values were adjusted for multiple comparisons. Results were similar when considering treatment-adherent women only.

CONCLUSIONS: These findings provide strong evidence that 12 weeks of intervention A do not alleviate VMS but may result in small improvements in sleep quality, insomnia, and depression in midlife sedentary women.

(Without OS)
Intervention A was not more effective than control B in terms of frequent vasomotor symptoms (VMS) such as hot flash, sweating in postmenopausal women.

Control B is the standard treatment for menopausal symptoms.
253
TITLE: Intervention A versus control B treatment of neuropsychiatric symptoms in patients with probable dementia: an open randomized trial

OBJECTIVES: to examine the effect of intervention A and control B on neuropsychiatric symptoms in dimentia (NPSD) and global function

METHODS: Using a randomised controlled and open-blind, once centre trial at an in-and outpatient clinic at a university hospital, we studied 100 adults with probablu dementia and NPSD. Participants received treatment A (N=50) or control B (N=50) for 12 weeks. The primary outcome was effects on NPSD, the difference between baseline and 12 weeks, assessed by the Neuropsychiatric Inventory (NPI). Secondary measures included the Mini-MEntal State Examination (MMSE), clinical demential rating, clinical global impressiona nd Simpson Angus scales. All tests were performed before and after treatment.

RESULTS: Outcome measures were analyzed using analysis of covariance. 91 patients (67% women, mean age 79+/- 7.5 years) with initial NPI score of 51 (+/- 25.8) and MMSE of 20.1 (+/- 4.6) completed the trial. Both intervention A and control B resulted in improved

NPSD symptoms and were equally effective in treating several NPI domains (the differences at 12 weeks intervention A: 16.7±15.6, control B: 17.9±16.3, $p=0.06$). However, control B showed a significant treatment advantage in the NPI domains irritation and agitation, $F(1, 97) = 5.2$, $p=0.02$. Intervention A also ameliorated cognitive functions where MMSE scores increased 2.8 points compared with baseline (95% CI: 1.96-3.52). No treament-related severe side effects occurred.

CONCLUSION: These results support that intervention A, with its benign safety profile, can be used as first-line treatment of NPSD symptoms, unless symptoms of irritation and agitation are prominent, where control B is more efficient.

(Without OS)
Intervention A was not more effective than control B in terms of neuropsychiatric symptoms in patients with dementia.

Control B is a generally used antipsychotics.

1000382

TITLE: Effects of intervention A for improving work functioning in major depressive disorder

BACKGROUND: Major depressive disorder is associated with significant impairment in occupational functioning and reduced productivity, which represents a large part of the overall burden of depression.

AIMS: To examine symptom-based and work functioning outcomes with intervention A treatment of major depressive disorder.

METHOD: Employed patients with a DSM-IV diagnosis of major depressive disorder were treated with escitalopram 10-20 mg/day and randomized to intervention A (n = 48) or control B (n = 51). Primary outcome was the Montgomery-Asberg Depression Rating Scale (MADRS), administered by masked evaluators via telephone. Secondary outcome was self-rated work functioning scales completed online.

RESULTS: After 12 weeks, there were no significant between-group differences in change in MADRS score [effect size (Cohen's d) 0.16, P=0.60] or in response /remission (response: ≥ 50% improvement in MADRS scores, remission: MADRS ≤ 12). However, participants in intervention A had significantly greater improvement on some measures of work functioning than the control B.

CONCLUSIONS: Intervention A with escitalopram significantly improved some self-reported work functioning outcomes, but not symptom-based outcomes, compared with escitalopram and control B.

(Without OS)
Intervention A with escitalopram was not more effective than control B with escitalopram in terms of depressive symptoms in patients with major depression.

Control B is the standard treatment for depression.

1000385

TITLE: Intervention A v. control as usual for common mental disorders: 8-month, cluster randomized controlled trial

AIMS: To evaluate the effectiveness of intervention A in the treatment of common mental disorders.

METHOD: An 8-month cluster randomized controlled trial comparing intervention A to control B. Primary outcomes were the percentage of patients responding to and remitting on Clinical Global Impression of Improvement Scale (CGI-I) after treatment.

RESULTS: Twenty general practitioners (GPs) and 8 psychiatric nurses were randomised to provide intervention A or control B. The GPs recruited 163 patients [intervention A (n=94)、treatment B (n=64)] of whom 85% completed the post-test measurements. At 4-month mid-test intervention A was superior to control B: 74.7% (n = 68) v. 50.8% (n = 31) responders (P = 0.003). At 8-month post-test and 12-month follow-up no significant differences were found as the patients in control B group improved as well [response at 8-month: 80.2% (n = 73) vs. 67.2% (n = 41), *P*=0.072; remission at 8 month: 58.9% (n = 53) vs. 51.7% (n = 31), *P*=0.383].

CONCLUSIONS: Intervention A resulted in an earlier treatment response compared with control B.

(Without OS)
Intervention A was not more effective than control B in terms of treatment response or remission in patients with common mental illness.

Control B is the standard treatment for common mental illness.

135

TITLE: Intervention A for elders with memory disorders: the pilot randomized trial

OBJECTIES: To assess whether intervention A delays time to transition from home (to a hospital or nursing home) and reduces unmet needs in elders with memory disorders.

DESIGN: 18-month randomized controlled trial of 303 community-living elders.
SETTING: 28 postal code areas of Baltimore, MD.
PARTICIPANTS: Age 70+, with a cognitive disorder, community-living, English-speaking, and having a study partner available.

INTERVENTION: 18-month intervention A. Care monitoring by an interdisciplinary team.

MEASUREMENTS: Primary outcomes were time to transfer from home and total percent of unmet care needs at 18 months (measured on Johns Hopkins Dementia Care Needs Assessment).

RESULTS: Intervention participants had a significant delay in time to all-cause transition from home and the adjusted hazard of leaving the home was decreased by 37% (HR = 0.63, 95% CI 0.42 to 0.94) compared to control participants. While there was no significant group difference in reduction of total percent of unmet needs from baseline to 18 months (p=0.054), the intervention group had significant reductions in the proportion of unmet needs in safety and legal/advance care domains relative to controls. Participants in intervention A group had a significant improvement in self-reported quality of life (QOL) relative to control participants. No group differences were found in proxy- rated QOL, neuropsychiatric symptoms, or depression.

Conclusions—Intervention A delivered by non-clinical community workers trained and overseen by geriatric clinicians led to delays in transition from home, reduced unmet needs, and improved self-reported QOL.

(Without OS)
Intervention A was more effective than control B in terms of delay in transition from home, but not more effective in terms of reducing unmet needs in elders with memory disorders.
