# Supplementary material for: Physician characteristics associated with proper assessment of overstated conclusions in research abstracts: A secondary analysis of a randomized controlled trial
Source: PLoS One. 2019 Jan 25;14(1):e0211206. doi: 10.1371/journal.pone.0211206 (PMC6347200; doi:10.1371/journal.pone.0211206)
Supplement: S1 Table — (PDF) [file pone.0211206.s002.pdf]

S1 Table. Physician characteristics classified by proper assessment of conclusion without overstatement

| Category | Characteristics | Proper[†] (n = 165) | | Not proper[‡] (n = 116) | | *p*-value[*] |
|---|---|---|---|---|---|---|
| Sex, n (%) | Male | 143 | ( 86.7 ) | 98 | ( 84.5 ) | 0.61 |
| Postgraduate year, mean (SD) | | 18.1 | ( 10.0 ) | 18 | ( 9.9 ) | 0.96 |
| Workplace, n (%) | Clinic | 62 | ( 37.6 ) | 42 | ( 36.2 ) | 0.81 |
| Board certification[§], n (%) | | 104 | ( 63.0 ) | 81 | ( 69.8 ) | 0.24 |
| Doctorate grade, n (%) | | 53 | ( 32.1 ) | 36 | ( 31.0 ) | 0.85 |
| PI[‖], n (%) | | 47 | ( 28.5 ) | 47 | ( 40.5 ) | 0.035 |
| Information resource, n (%) | Pharmacological company[§] | 18 | ( 10.9 ) | 10 | ( 8.6 ) | 0.53 |
| EBM workshop[**], n (%) | | 104 | ( 63.0 ) | 77 | ( 66.4 ) | 0.56 |
| Abstract ≥5[††], n (%) | | 66 | ( 40.0 ) | 63 | ( 54.3 ) | 0.018 |

[*] *p*-value for Fisher's Exact test

[†] Rating 5 or more for the validity of overstated abstract conclusion on a scale of 0 to 10, with 0 being not at all and 10 being very likely

[‡] Rating less than 5 for the validity of overstated abstract conclusion on a scale of 0 to 10, with 0 being not at all and 10 being very likely

[§] Any board certification

[‖] Clinical research experience as a principal investigator

[¶] Access to research information (only from pharmacological company)

[**] Ever attended an evidence based medicine workshop

[††] Reading 5 or more abstracts in the last month

SD= Standerdised deviation, PI = Principal investigatorr, EBM =Evidence based medicine
